# Neural Progenitors in the Developing Neocortex of the Northern Tree Shrew (*Tupaia belangeri*) Show a Closer Relationship to Gyrencephalic Primates Than to Lissencephalic Rodents

**DOI:** 10.3389/fnana.2018.00029

**Published:** 2018-04-19

**Authors:** Sebastian Römer, Hannah Bender, Wolfgang Knabe, Elke Zimmermann, Rudolf Rübsamen, Johannes Seeger, Simone A. Fietz

**Affiliations:** ^1^Institute of Veterinary Anatomy, Histology and Embryology, University of Leipzig, Leipzig, Germany; ^2^Prosektur Anatomie, Medizinische Fakultät, Westfälische Wilhelms-Universität Münster, Münster, Germany; ^3^Institute of Zoology, University of Veterinary Medicine Hanover, Hanover, Germany; ^4^Institute of Biology, Faculty of Biosciences, Pharmacy and Psychology, University of Leipzig, Leipzig, Germany

**Keywords:** neocortex development, neural progenitor, basal radial glia, tree shrew, *Tupaia belangeri*

## Abstract

The neocortex is the most complex part of the mammalian brain and as such it has undergone tremendous expansion during evolution, especially in primates. The majority of neocortical neurons originate from distinct neural stem and progenitor cells (NPCs) located in the ventricular and subventricular zone (SVZ). Previous studies revealed that the SVZ thickness as well as the abundance and distribution of NPCs, especially that of basal radial glia (bRG), differ markedly between the lissencephalic rodent and gyrencephalic primate neocortex. The northern tree shrew (*Tupaia belangeri*) is a rat-sized mammal with a high brain to body mass ratio, which stands phylogenetically mid-way between rodents and primates. Our study provides – for the first time – detailed data on the presence, abundance and distribution of bRG and other distinct NPCs in the developing neocortex of the northern tree shrew (*Tupaia belangeri*). We show that the developing tree shrew neocortex is characterized by an expanded SVZ, a high abundance of Pax6+ NPCs in the SVZ, and a relatively high percentage of bRG at peak of upper-layer neurogenesis. We further demonstrate that key features of tree shrew neocortex development, e.g., the presence, abundance and distribution of distinct NPCs, are closer related to those of gyrencephalic primates than to those of ferret and lissencephalic rodents. Together, our study provides novel insight into the evolution of bRG and other distinct NPCs in the neocortex development of Euarchontoglires and introduces the tree shrew as a potential novel model organism in the area of human brain development and developmental disorders.

## Introduction

The neocortex is the most complex part of the mammalian brain and has undergone tremendous expansion during evolution, especially in the primate lineage. The overwhelming majority of neocortical neurons are generated during embryonic and fetal development. They mostly originate from neural stem and progenitor cells (NPCs), which are characterized by distinct cell biological features and located in the two germinal zones: ventricular (VZ) and subventricular zone (SVZ) (Götz and Huttner, 2005; Fietz and Huttner, 2011; LaMonica et al., 2012; Florio and Huttner, 2014; De Juan Romero and Borrell, 2015; Montiel et al., 2016; Namba and Huttner, 2017). The VZ, the most apical layer of the cortical wall, contains the cell bodies of the primary NPCs, the apical progenitors (APs). APs consist of neuroepithelial cells (NECs) that transform into apical radial glia (aRG) at the onset of neurogenesis (Kriegstein and Götz, 2003; Götz and Huttner, 2005) and the apical intermediate progenitors (aIPs), also known as short neural precursors (Gal et al., 2006; Stancik et al., 2010). All three subtypes have an apical domain, which consists of an apical plasma membrane, an apical cell cortex and apical adherens junctions and predominantly maintain a radially oriented process, which spans the entire neocortical wall throughout the cell cycle in NECs and aRG, and retracts from the basal lamina for mitosis in aIPs (Rakic, 1972; Aaku-Saraste et al., 1997; Chenn et al., 1998; Götz and Huttner, 2005; Gal et al., 2006; Marthiens and Ffrench-Constant, 2009). Before the onset of neurogenesis, NECs mainly undergo symmetric proliferative divisions, resulting in the lateral expansion of the VZ (Rakic, 1995). With the onset of neurogenesis, aIPs mostly undergo symmetric neurogenic divisions, while aRG start dividing asymmetrically, thereby giving rise to the secondary NPCs, the basal progenitors (BPs) that accumulate in the SVZ, basal to the VZ (Haubensak et al., 2004; Miyata et al., 2004; Noctor et al., 2004; Stancik et al., 2010). BPs comprise of two major subtypes, the basal intermediate progenitors (bIPs) and basal radial glia (bRG), the latter also referred to as outer RG cells (Hansen et al., 2010; Wang et al., 2011), intermediate RG cells (Reillo et al., 2011) or translocating RG cells (Martinez-Cerdeno et al., 2012). Both BP subtypes lack an apical domain; however, whereas bIPs retract their processes prior to M-phase, bRG maintain at least one radially oriented process throughout the cell cycle with a subset of them reaching up to the basal lamina (Attardo et al., 2008; Fietz et al., 2010; Hansen et al., 2010; Reillo et al., 2011; Nowakowski et al., 2016). Depending on the number and orientation of processes, three different bRG morphotypes have been identified (Betizeau et al., 2013): (1) bRG with a basal process, referred to as bRG-basal-process (-P), (2) bRG with an apical process, referred to as bRG-apical-P and (3) bRG with a basal and an apical process, referred to as bRG-both-P.

Although the SVZ is regarded as the developmental milestone of a six-layered neocortex, its thickness varies considerably between species showing different degrees of neocortex expansion (Smart et al., 2002; Cheung et al., 2010; Fietz et al., 2010; Reillo et al., 2011; Garcia-Moreno et al., 2012; Kelava et al., 2012; Martinez-Cerdeno et al., 2012, 2017; Sauerland et al., 2018). In particular, the SVZ of primates, that possess an extremely high degree of neocortex expansion, is substantially thicker at peak stages of neurogenesis when compared to non-primate species (Smart et al., 2002; Martinez-Cerdeno et al., 2012). Besides its increase in thickness, the SVZ of primates and other mammals possessing a relatively expanded neocortex such as ferret, cat, sheep and agouti is clearly subdivided into two morphologically distinct germinal zones: an inner SVZ (iSVZ), which largely resembles mouse SVZ, and an outer SVZ (oSVZ) which is absent in mouse or relatively thin in rat (Smart et al., 2002; Fietz et al., 2010; Hansen et al., 2010; Reillo et al., 2011; Garcia-Moreno et al., 2012; Martinez-Cerdeno et al., 2012). The increase and remodeling of the SVZ is accompanied by significant changes in its BP subtype composition, mainly affecting their abundance rather than their occurrence. As such, bRG have been shown to occur in the developing neocortex of species from various mammalian orders and infraclasses (Fietz et al., 2010; Hansen et al., 2010; Reillo et al., 2011; Wang et al., 2011; Martinez-Cerdeno et al., 2012, 2017; Sauerland et al., 2018). However, its relative abundance varies substantially between species showing different degrees of neocortex expansion. In mice, the SVZ mostly contains bIPs and only a minor fraction of bRG (∼10%), whereas its abundance increases in the SVZ of sheep, ferret, marmoset and tammar wallaby (∼30%) and peak in the SVZ, especially the oSVZ, of gyrencephalic primates, in which bRG become the most abundant BP type (>50%) (Fietz et al., 2010; Hansen et al., 2010; Reillo et al., 2011; Kelava et al., 2012; Betizeau et al., 2013; Pilz et al., 2013; Florio and Huttner, 2014; Sauerland et al., 2018). Importantly, the overwhelming majority of BPs, i.e., bIPs, in lissencephalic rodents undergoes symmetric proliferative divisions and thus shows limited proliferative potential, whereas a major fraction of BPs, i.e., bRG, in gyrencephalic primates is able to undergo repeated cell division, thereby displaying high proliferative potential (Haubensak et al., 2004; Miyata et al., 2004; Noctor et al., 2004; Attardo et al., 2008; Hansen et al., 2010; Betizeau et al., 2013). This increased abundance in proliferative BPs results in the radial expansion of the SVZ, notably the oSVZ, which ultimately promotes a higher neuronal output in gyrencephalic primates, particularly in human (Fietz et al., 2010; Hansen et al., 2010; Betizeau et al., 2013; Gertz et al., 2014).

The northern tree shrew (*Tupaia belangeri*) is a rat-sized mammal with an average gestation period of 43.7 days (Kuhn and Schwaier, 1973) and a close phylogenetic relationship to primates and rodents (Supplementary Figure 1). It belongs to the separate order Scandentia which is currently grouped with primates and culogos (flying lemurs) within the grandorder Euarchonta, a sister group of Glires consisting of rodents and lagomorphs (Janecka et al., 2007; Song et al., 2012; Kumar et al., 2013). Recent genome studies suggested that the Chinese tree shrew (*Tupaia belangeri chinensis*) possesses a closer genetic relationship to primates than to rodents (Supplementary Figure 1A) (Fan et al., 2013; Lin et al., 2014). Although the tree shrew exhibits – comparable to most rodents - a lissencephalic neocortex; it has, however, a high brain to body mass ratio and shows neuroanatomical characteristics that are highly similar to those of primates including the cytoarchitecture and organization of the somatosensory, visual and motor cortex (Supplementary Figure 1B) (Sur et al., 1980; Elston et al., 2005; Remple et al., 2006, 2007; Wong and Kaas, 2009b; Veit et al., 2011). Until now, precise data on the presence, abundance and distribution of bRG and other distinct NPCs in the developing tree shrew neocortex are lacking. It was therefore the aim of this study to characterize and quantify the germinal zones and containing NPCs in the developing neocortex of the northern tree shrew (*Tupaia belangeri*). Moreover, by comparing our results with published data from phylogenetically closely related species, this study aims to answer the question whether key features of tree shrew neocortex development, e.g., the presence, abundance and distribution of the distinct NPCs, show a closer relationship to gyrencephalic primates or to lissencephalic rodents.

## Materials and Methods

### Brain Samples

**FIGURE 1:**
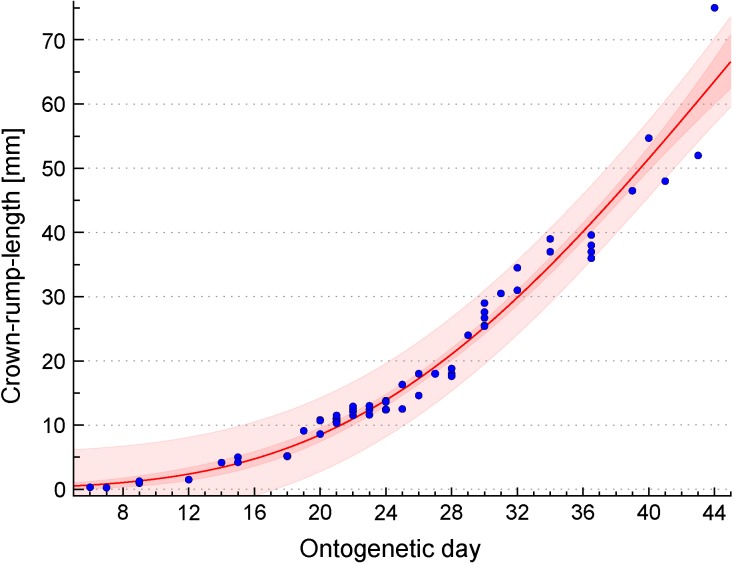
Developmental growth curve of *Tupaia belangeri*. Plot of the relationship between ontogenetic day and crown-rump length (CRL) which has been determined in 67 *Tupaia belangeri* embryos. The CRL growth curve (red line) was estimated by the following Gompertz function: ontogenetic day = 197.918^∗^exp[–exp(1.997 – 0.043^∗^CRL)]. Dark red and light red areas represent the 95% confidence band and the 95% prediction band, respectively. For details, see Section “Materials and Methods.”

*Tupaia belangeri* brain tissue was received from the animal facilities, Institute of Biology, Faculty of Biosciences, Pharmacy, and Psychology, University of Leipzig and Institute of Zoology, University for Veterinary Medicine Hannover. Animals were housed in 125 cm × 100 cm × 80 cm wire mesh cages. Temperature averaged 22°C, humidity was kept at approximately 55% and light/dark cycle was set to 12:12. Water and standard diet pellets (Altromin Spezialfutter GmbH and Co. KG, Lage, Germany) was given *ad libitum* supplemented by fresh fruits, mealworms and locusts. Breeding pairs were not closely related to each other and pair partners were permanently housed together. Successful mating was indicated by a change in the female receptive behavior toward the male. Successful pregnancy was determined based on body weight gain. Animals were anesthetized by an intraperitoneal overdose of pentobarbital (200 mg/kg). The age of the animals ranged from embryonic/fetal day (E) 32 (*n* = 2), 37 (*n* = 2), 45 (*n* = 2) to postnatal day (P) 1 (*n* = 2) and was determined with the help of the crown-rump-length (CRL) growth curve described below (**Figure 1**). All experiments were performed in accordance with German animal welfare legislation and were approved by the Landesdirektion Leipzig. Embryos/fetuses and neonates were carefully dissected, brains were fixed immediately in 4% paraformaldehyde (PFA) for at least 2 days and stored in PBS at 4°C until processing.

### Determination of the *Tupaia belangeri* Growth Curve

Crown-rump-lengths were provided for 57 embryos/fetuses of *Tupaia belangeri*, all belonging to the tree shrew collection of H.-J. Kuhn (Senckenberg Museum, Frankfurt/Main, Germany; present location: Department of Prosektur Anatomie, Westfälische Wilhelms-Universität Münster, Münster, Germany). Detailed descriptions of the collection including preparation procedures have been published previously (Knabe and Kuhn, 1998; Washausen et al., 2005; Knabe et al., 2007, 2008; Knabe and Washausen, 2015). In brief, pregnant females were obtained from the Battelle Institute (Frankfurt/Main, Germany) and from the German Primate Center (Göttingen, Germany) (former Sonderforschungsbereich 89, Cardiology, 1976–1982). Given that in *Tupaia belangeri* ovulation is induced by copulation, the day of fertilization was considered as ontogenetic day 1. For the construction of the growth curve (**Figure 1**), 10 additional CRL measurements were included (Kuhn and Schwaier, 1973). Using CurveExpert Professional (Hyams Development, Chattanooga, TN, United States), the CRL growth curve was estimated by the following Gompertz function: ontogenetic day = 197.918^∗^exp[-exp(1.997 - 0.043^∗^CRL)].

### Immunocytochemistry

Brain samples were processed and subjected to an immunohistochemistry protocol as described previously (Sauerland et al., 2018). In brief, brains were incubated in 30% sucrose in PBS at room temperature, embedded in Tissue-Tek (Sakura Finetek) and stored at -20°C. Cryosections were cut at 30 μm and kept at -20°C. Complete telencephalon was cut coronally and sections at a medium position with regard to the rostro-caudal axis were used for immunohistochemistry and heated for 1 h at 90–95°C in 0.01 M citrate buffer (pH 6), permeabilized with 0.3% Triton X-100 in PBS and quenched with 0.1 M glycine. Primary antibodies were incubated overnight at 4°C. The following primary antibodies were used: Tbr1 (1:200, rabbit, Millipore, AB 10554), Pax6 (1:100, rabbit, Biozol, BLD 901301), Tbr2 (1:100, sheep, R&D Systems, AF61669), PCNA (1:100, mouse, Novus Biologicals, NB500-106), phosphorylated vimentin (pVim, 1:100, mouse, Abcam, ab22651), Par3 (1:200, rabbit, Millipore, 07-330), ZO-1 (1:200, mouse, Invitrogen, 33-9100), Tau1 (1:100, mouse, Millipore, MAB3420), Brn2 (1:100, mouse, Santa Cruz, sc-393324). Donkey secondary antibodies coupled to Alexa 488, 555, and 647 (1:500, Life Technologies) were incubated for 1 h at room temperature. All sections were counterstained with DAPI (1:500, Sigma), mounted in Mowiol (Merck Biosciences) and kept at 4°C.

### Image Acquisition and Analysis

**FIGURE 2:**
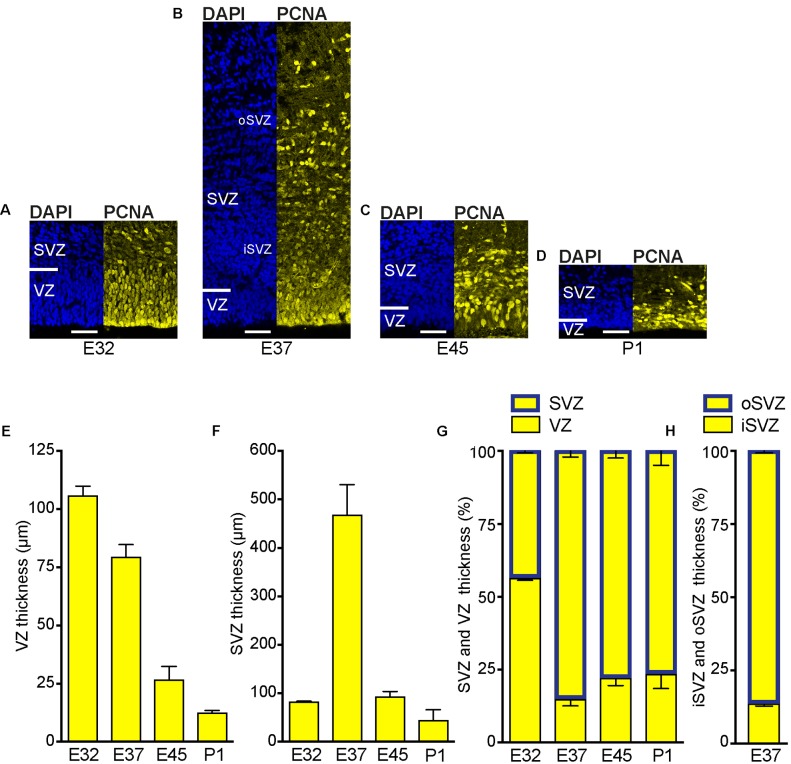
Development of the germinal zones in the tree shrew neocortex. **(A–D)** Immunofluorescence for PCNA (yellow) and DAPI staining (blue) on 30 μm-cryosections of E32–P1 tree shrew neocortex. The top margin of the image corresponds to the transition zone SVZ/intermediate zone. Scale bars, 50 μm. VZ, ventricular zone; SVZ, subventricular zone; iSVZ, inner SVZ; oSVZ, outer SVZ. **(E)** Quantification of the VZ thickness of the E32–P1 tree shrew neocortex. **(F)** Quantification of the SVZ thickness of the E32–P1 tree shrew neocortex. **(G)** Quantification of the VZ and SVZ thickness of the E32–P1 tree shrew neocortex, expressed as percentage of the sum of VZ and SVZ. **(H)** Quantification of the iSVZ and oSVZ thickness of the E37 tree shrew neocortex, expressed as percentage of the sum of iSVZ and oSVZ. **(E–H)** Data represent mean ± SD and were obtained from two consecutive sections of two brains each.

**FIGURE 3:**
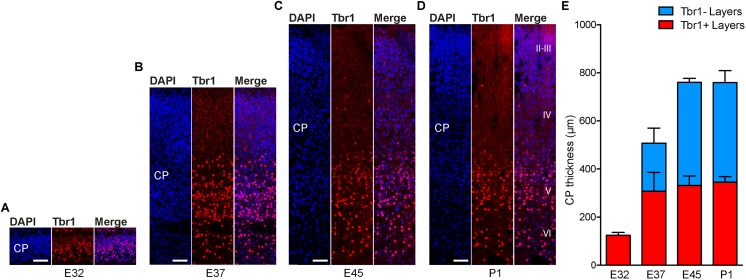
Cortical plate development in the tree shrew neocortex. **(A–D)** Immunofluorescence for Tbr1 (red) and DAPI staining (blue) on 30 μm-cryosections of E32–P1 tree shrew neocortex. The bottom margin corresponds to the transition zone CP/subplate. Cortical layers II–VI are indicated in **(D)**. Scale bars, 50 μm. CP, cortical plate. **(E)** Quantification of the thickness of Tbr1+ and Tbr1– CP layers in the E32–P1 tree shrew neocortex. Data represent mean ± SD and were obtained from consecutive two sections of two brains each.

**FIGURE 4:**
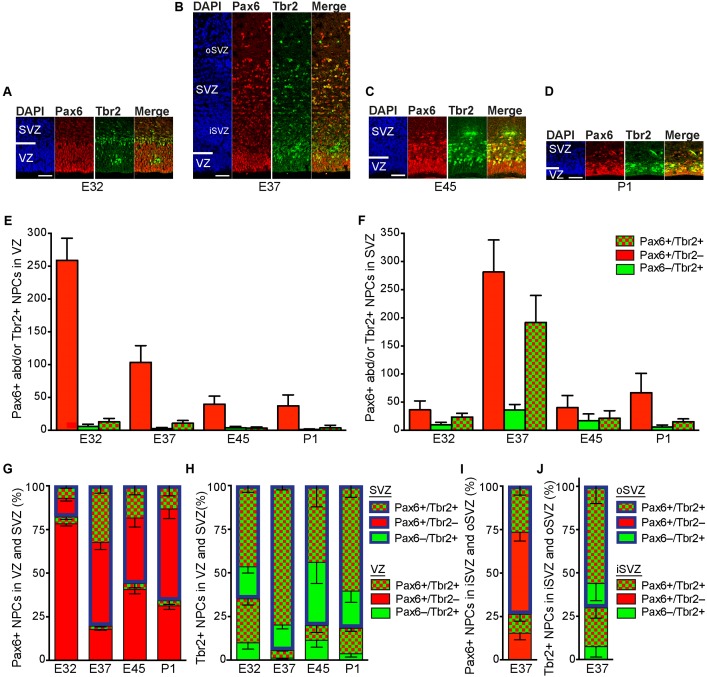
Pax6 and Tbr2 expression in the germinal zones of the developing tree shrew neocortex. **(A–D)** Double-Immunofluorescence for Pax6 (red) and Tbr2 (green) and DAPI staining (blue) on 30 μm-cryosections of E32–P1 tree shrew neocortex. The top margin of the image corresponds to the transition zone SVZ/intermediate zone. Scale bars, 50 μm. VZ, ventricular zone; SVZ, subventricular zone; iSVZ, inner SVZ; oSVZ, outer SVZ. **(E,F)** Quantification of Pax6+/Tbr2–, Pax6+/Tbr2+ and Pax6–/Tbr2+ NPCs in the VZ **(E)** and SVZ **(F)** of the E32–P1 tree shrew neocortex, expressed as number of cells per 200 μm ventricular surface. Color legend is shown in **(F)**. **(G)** Quantification of Pax6+ (Pax6+/Tbr2–, Pax6+/Tbr2+) NPCs **(G)** and Tbr2+ (Pax6–/Tbr2+, Pax6+/Tbr2+) NPCs **(H)** in the VZ and SVZ of the E32–P1 tree shrew neocortex, expressed as percentage of the sum of VZ and SVZ. Color legend is shown in **(H)**. **(I,J)** Quantification of Pax6+ (Pax6+/Tbr2–, Pax6+/Tbr2+) NPCs **(I)** and Tbr2+ (Pax6–/Tbr2+, Pax6+/Tbr2+) NPCs **(J)** in the iSVZ and oSVZ of the E32–P1 tree shrew neocortex, expressed as percentage of the sum of iSVZ and oSVZ. Color legend is shown in **(J)**. **(E–J)** Cortical wall corresponding to a total ventricular surface of 4.12–12.081 mm was analyzed. Data represent mean ± SD and are from two brains each.

**FIGURE 5:**
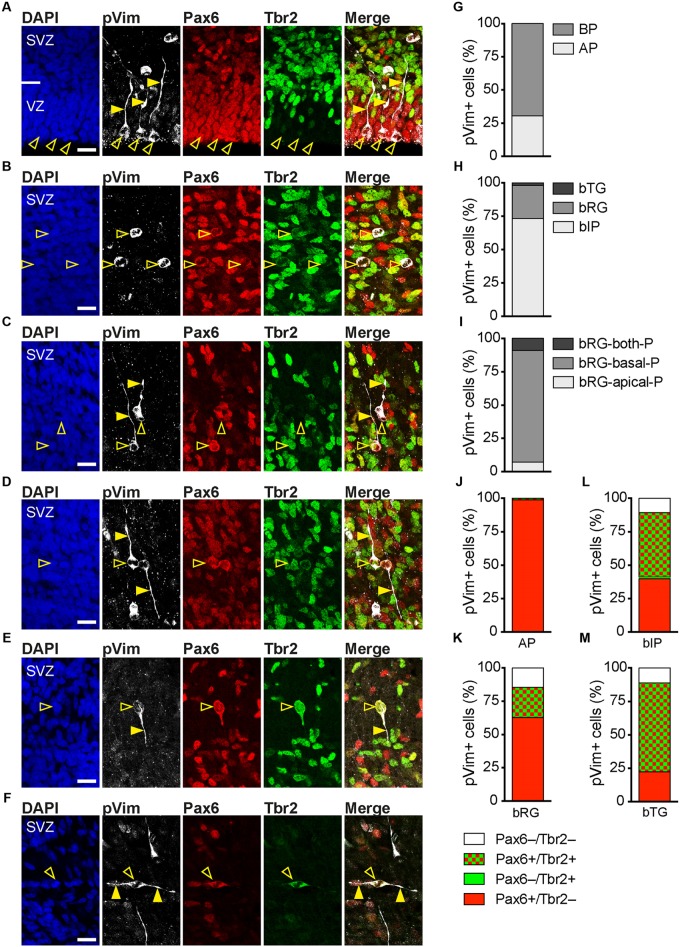
Neural progenitor subtypes in the developing tree shrew neocortex. **(A–F)** Triple-immunofluorescence for phospho-vimentin (pVim, white), Pax6 (red), and Tbr2 (green) and DAPI staining (blue) on 30 μm-cryosections of E37 tree shrew neocortex. The merge images show combined immunofluorescence for pVim, Pax6, and Tbr2. Open arrowheads, cell bodies; solid arrowheads, pVim+ processes. Scale bars, 20 μm. VZ, ventricular zone; SVZ, subventricular zone. **(G–I)** Quantification of the distinct progenitor cell types at M-phase, identified by their location of mitosis and morphology at M-phase, in the E37 tree shrew neocortex, expressed as the percentage of total pVim+ cells. Data are the sum of two brains. The total number of pVim+ mitoses quantified was 642. **(J–M)** Quantification of the distinct progenitor cell types at M-phase, identified by pVim staining, that are Pax6+/Tbr2– (red), Pax6+/Tbr2+ (red/green), Pax6–/Tbr2+ (green), or Pax6–/Tbr2– (white) in the E37 tree shrew neocortex. Data are the sum of two brains. The total number of pVim+ mitoses analyzed was as in **(G–I)**. **(G–M)** AP, apical progenitor; BP, basal progenitor; bIP, basal intermediate progenitor; bRG, basal radial glia, bTG; basal tangential glia; -P, process.

**FIGURE 6:**
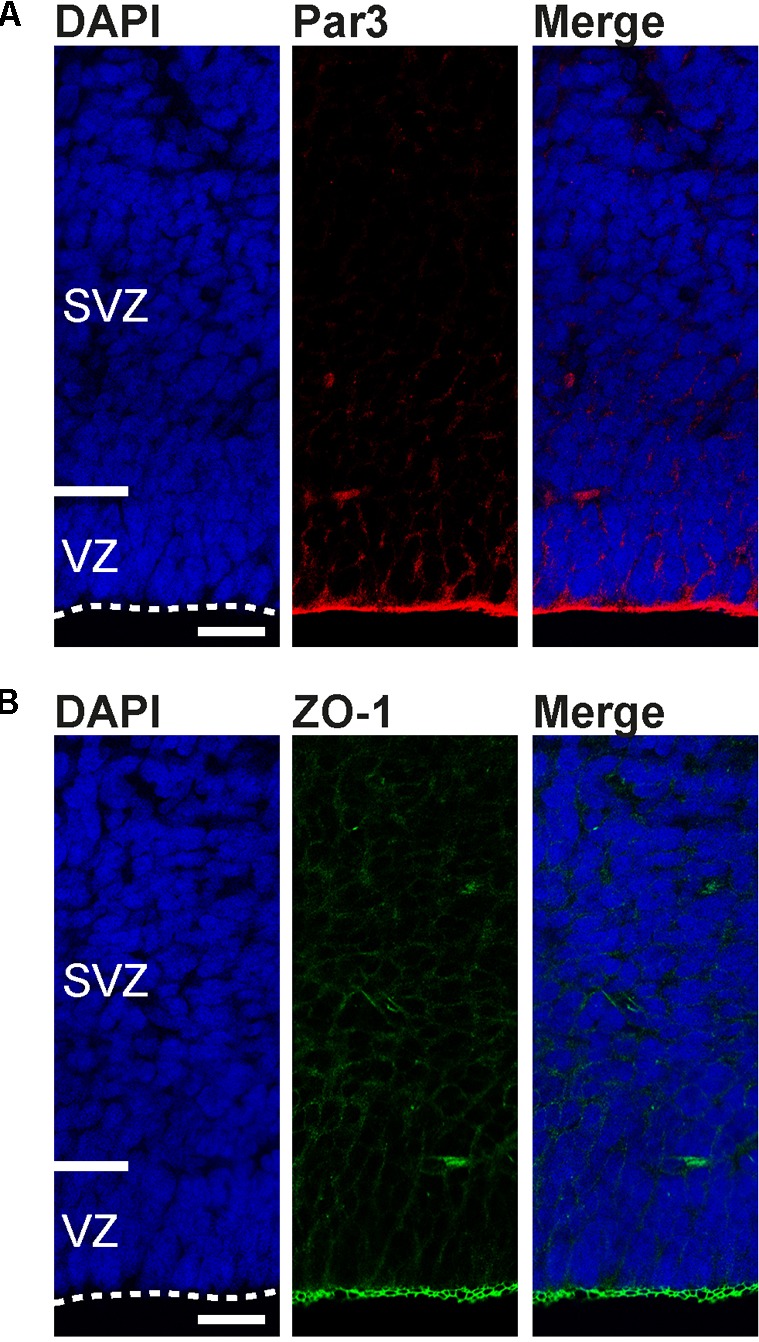
Apical domain markers are concentrated at the developing tree shrew ventricular surface. **(A,B)** Immunofluorescence for Par3 (A, red), ZO-1 (B, green), and DAPI staining (blue) on 30 μm-cryosections of E37 tree shrew neocortex. Dashed lines indicate the ventricular surface. Scale bars, 50 μm. VZ, ventricular zone. SVZ, subventricular zone.

Fluorescence images were acquired with a Leica SP8 confocal laser-scanning microscope, using a 20× or 40× objective. Images were taken as 3.121 μm (20×) or 1.271 μm (40×) single optical sections and processed using Fiji and Photoshop (Adobe) software. Panels of **Figures 1**–**4**, **6** represent single optical sections. Panels of **Figure 5** represent stacks of 3–5 optical sections. The VZ, SVZ, intermediate zone (IZ)/subplate (SP) and cortical plate (CP) were identified based on their cytoarchitecture as described previously (Sauerland et al., 2018). In brief, the VZ appeared as a densely packed cell layer that lines the lateral ventricle and whose nuclei exhibit radial morphology. The SVZ was identified as a cell layer adjacent to the VZ that exhibits a looser and sparser cell arrangement than the VZ. The IZ/SP was identified as a cell layer that exhibits a very low cell density between the SVZ and the CP. The CP was identified as densely packed cell layer adjacent to the IZ/SP. To compare data across species, iSVZ and oSVZ were identified based on previously established criteria (Smart et al., 2002; Martinez-Cerdeno et al., 2012). Specifically, the iSVZ was identified as the inner SVZ cell layer that exhibits a higher cell density and a more random cell organization than the oSVZ. The iSVZ corresponded to the dense inner band of Tbr2+ cells, which was localized to the Tau1-free zone surrounding the lateral ventricle (Supplementary Figure 2). The oSVZ was identified as the outer SVZ cell layer that exhibits a relatively loose and sparse cell arrangement and whose nuclei mostly exhibit radial morphology. The oSVZ corresponded to the diffuse outer band of Tbr2+ cells, which was localized to the Tau1-striated zone (Supplementary Figure 2).

Quantification of cells was performed with Fiji software using a Multiclass Cell Counter plug in Schindelin et al. (2012) and applying a default threshold of 100/255 on dark background. All quantifications were performed on images from the dorsolateral telencephalon. The radial thickness of the germinal zones and CP as well as the length of the ventricular surface were determined using Fiji software. Data were further processed using Prism software.

### Statistical Analysis of the Relationship Between Tree Shrew, Macaque, Ferret, and Rat/Mouse Neocortex Development

Statistical analysis of the relationship between tree shrew, macaque, ferret and rat/mouse neocortex development was conducted in R. Parameters of macaque, ferret and rat/mouse neocortex development at peak of deep-layer and upper-layer neurogenesis were obtained from the literature (**Table 1**). Due to the fact that not all parameters were available from a single rodent species, data of rat and mouse neocortex development were used, i.e., neurodevelopmental parameters 1–12 were obtained from rat (Sprague Dawley rat) and neurodevelopmental parameters 13–15 were obtained from mouse (Swiss Webster mouse). Parameters of *Tupaia belangeri* neocortex development at peak of deep-layer and upper-layer neurogenesis were analyzed in this study. Neurodevelopmental parameters 1, 2, 4–12 were obtained from a similar cortical area, predominantly representing somatosensory cortex. In case of neurodevelopmental parameters 3, 13–15, tree shrew and ferret data were obtained from a cortical area, predominantly representing somatosensory cortex, and macaque data were obtained from the visual cortex. The thicknesses of the germinal zones (neurodevelopmental parameters 3, 4) were measured using the images obtained from the literature (**Table 1**). Taken into account that different protocols were used for the quantification of cells in the developing tree shrew, macaque, ferret and rat neocortex and thus to ensure data comparability, relative values (ratios) of absolute Pax6+ NPC, Tbr2+ NPC and mitotic NPC counts between SVZ and VZ (neurodevelopmental parameters 6–12) were calculated from the absolute values and used in the analysis (**Table 2**).

**Table 1:** List of references of neurodevelopmental parameters used for statistical analysis.

Neurodevelopmental parameter		Rat/Mouse	Ferret	Macaque
1	SVZ thickness at DL neurogenesis (μm)	Table 5 (Martinez-Cerdeno et al., 2012)	Table 5 (Martinez-Cerdeno et al., 2012)	Table 5 (Martinez-Cerdeno et al., 2012)
2	SVZ thickness at UL neurogenesis (μm)	Table 5 (Martinez-Cerdeno et al., 2012)	Table 5 (Martinez-Cerdeno et al., 2012)	Table 5 (Martinez-Cerdeno et al., 2012)
3	Ratio of SVZ/VZ thickness at DL neurogenesis	Figure 1A (Ida et al., 2006)	Figure 1O (Fietz et al., 2010)	Figure 1A (Betizeau et al., 2013)
4	Ratio of SVZ/VZ thickness at UL neurogenesis	Figure 2B (Martinez-Cerdeno et al., 2006)	Figure 1Q (Fietz et al., 2010)	Figure 2A (Martinez-Cerdeno et al., 2012)
5	Ratio of oSVZ/iSVZ thickness at UL neurogenesis	Table 5 (Martinez-Cerdeno et al., 2012)	Table 5 (Martinez-Cerdeno et al., 2012)	Table 5 (Martinez-Cerdeno et al., 2012)
6	Ratio of Tbr2+ NPCs in SVZ/VZ at DL neurogenesis	Table 4 (Martinez-Cerdeno et al., 2012)	Table 4 (Martinez-Cerdeno et al., 2012)	Table 4 (Martinez-Cerdeno et al., 2012)
7	Ratio of Tbr2+ NPCs in SVZ/VZ at UL neurogenesis	Table 4 (Martinez-Cerdeno et al., 2012)	Table 4 (Martinez-Cerdeno et al., 2012)	Table 4 (Martinez-Cerdeno et al., 2012)
8	Ratio of Tbr2+ NPCs in oSVZ/iSVZ at UL neurogenesis	Table 4 (Martinez-Cerdeno et al., 2012)	Table 4 (Martinez-Cerdeno et al., 2012)	Table 4 (Martinez-Cerdeno et al., 2012)
9	Ratio of Pax6+ NPCs in SVZ/VZ at DL neurogenesis	Table 7 (Martinez-Cerdeno et al., 2012)	Table 7 (Martinez-Cerdeno et al., 2012)	Table 7 (Martinez-Cerdeno et al., 2012)
10	Ratio of Pax6+ NPCs in SVZ/VZ at UL neurogenesis	Table 7 (Martinez-Cerdeno et al., 2012)	Table 7 (Martinez-Cerdeno et al., 2012)	Table 7 (Martinez-Cerdeno et al., 2012)
11	Ratio of Pax6+ NPCs in oSVZ/iSVZ at UL neurogenesis	Table 7 (Martinez-Cerdeno et al., 2012)	Table 7 (Martinez-Cerdeno et al., 2012)	Table 7 (Martinez-Cerdeno et al., 2012)
12	Ratio of mitoses in SVZ/VZ	Table 8 (Martinez-Cerdeno et al., 2012)	Table 8 (Martinez-Cerdeno et al., 2012)	Table 8 (Martinez-Cerdeno et al., 2012)
13	Relative abundance of bRG at UL neurogenesis (%)	(Wang et al., 2011)	Figure 3L (Fietz et al., 2010)	Figure 4E (Betizeau et al., 2013)
14	Relative abundance of Pax6+ bRG at UL neurogenesis (%)	(Wang et al., 2011)	Figure 3O (Fietz et al., 2010)	Figure 3J (Betizeau et al., 2013)
15	Relative abundance of Tbr2+ bRG at UL neurogenesis (%)	Figure S8H (Florio et al., 2015)	Figure 3O (Fietz et al., 2010)	Figure 3J (Betizeau et al., 2013)


**Parameters of macaque, rat/mouse and ferret neocortex development were obtained from the literature as listed. VZ, ventricular zone; SVZ, subventricular zone; iSVZ, inner SVZ; oSVZ, outer SVZ; DL, deep-layer; UL, upper-layer. For details, see Section “Materials and Methods.”**

**Table 2:** Untransformed values of neurodevelopmental parameters of tree shrew, macaque, rat/mouse and ferret used for statistical analysis.

Neurodevelopmental parameter		Rat/Mouse	Ferret	Tree shrew	Macaque
1	SVZ thickness at DL neurogenesis (μm)	27	97	82.1	440
2	SVZ thickness at UL neurogenesis (μm)	237	545	467.034	2590
3	Ratio of SVZ/VZ thickness at DL neurogenesis	0.333	0.8	0.778	0.667
4	Ratio of SVZ/VZ thickness at UL neurogenesis	0.875	2.333	5.901	37.5
5	Ratio of oSVZ/iSVZ thickness at UL neurogenesis	0.975	1.148	6.436	4.18
6	Ratio of Tbr2+ NPCs in SVZ/VZ at DL neurogenesis	1.563	2.464	1.772	28.667
7	Ratio of Tbr2+ NPCs in SVZ/VZ at UL neurogenesis	1.196	4.581	16.813	39.4
8	Ratio of Tbr2+ NPCs in oSVZ/iSVZ at UL neurogenesis	0.134	0.331	2.337	1.736
9	Ratio of Pax6+ NPCs in SVZ/VZ at DL neurogenesis	0.104	0.056	0.219	0.125
10	Ratio of Pax6+ NPCs in SVZ/VZ at UL neurogenesis	0.138	0.697	4.131	3.85
11	Ratio of Pax6+ NPCs in oSVZ/iSVZ at UL neurogenesis	0.026	0.817	2.955	1.982
12	Ratio of mitoses in SVZ/VZ	0.678	1.529	2.276	1.788
13	Relative abundance of bRG at UL neurogenesis (%)	8.89	42	24.664	62
14	Relative abundance of Pax6+ bRG at UL neurogenesis (%)	100	100	86	91
15	Relative abundance of Tbr2+ bRG at UL neurogenesis (%)	75	0	23	44


**Neurodevelopmental parameters of tree shrew were analyzed in this study. All other parameters were obtained from the literature (**Table 1**). VZ, ventricular zone; SVZ, subventricular zone; iSVZ, inner SVZ; oSVZ, outer SVZ; DL, deep-layer; UL, upper-layer. For details, see section “Materials and Methods.”**

For rank comparison, untransformed values of distinct parameters of cortex development (**Table 2**) were used. A rank (1–4) was assigned within each parameter to each species and the average rank over all parameters for each species was calculated. Statistical differences between the average ranks of the different species were tested with the Kruskal–Wallis-test followed by Conover’s *post hoc* test using Holm correction (Pohlert, 2014). *P*-values below 0.05 were considered significant. For hierarchical clustering, principal component analysis (PCA) and Euclidean distance computation, untransformed values of distinct parameters of cortex development (**Table 2**) were transformed into *z*-scores (standard scores). Hierarchical clustering of species was performed based on Euclidean distance.

## Results

### SVZ Is Markedly Increased at Peak of Upper-Layer Neurogenesis in the Tree Shrew Neocortex

We first examined the development of the germinal zones in the tree shrew neocortex, and analyzed E32–P1 cortical sections by immunohistochemistry for PCNA, a specific marker for proliferating cells (**Figures 2A–D** and Supplementary Figure 3). At the earliest developmental stage analyzed, the VZ appeared as a prominent and tightly packed cell layer in which PCNA+ nuclei show radial morphology (**Figure 2A**). The SVZ, in which PCNA+ nuclei are more loosely arranged, was already present at that stage but appeared relatively thin (**Figure 2A**). Quantification of the thickness of the germinal zones revealed the VZ to be the predominant germinal zone at E32 (**Figures 2E–G**). After E32, the thickness of the VZ progressively declined (**Figures 2B–E**), whereas that of the SVZ rapidly increased, especially until E37, revealing the SVZ to become the major germinal zone at later stages of tree shrew neocortex development (**Figures 2B–D,F,G**). These findings are similar to the development of the germinal zones in the neocortex of gyrencephalic primates including human and other species exhibiting an expanded neocortex such as ferret, but are in contrast to that of lissencephalic rodents in which the VZ constitutes the major NPC pool throughout development (Smart et al., 2002; Kriegstein et al., 2006; Bayatti et al., 2008; Kriegstein and Alvarez-Buylla, 2009; Fietz et al., 2010; Hansen et al., 2010; Reillo et al., 2011; Martinez-Cerdeno et al., 2012). Interestingly, when a clear distinction between the iSVZ and oSVZ was detectable at E37, its thickness ratio was similar to that of macaque and clearly different when compared to that of rat and ferret (**Figure 2H**) (Martinez-Cerdeno et al., 2012).

To investigate whether the development of the germinal zones, especially the SVZ, and of the CP, show a similar time course, we next analyzed cortical sections by immunohistochemistry for Tbr1, that is characteristically expressed by early-born deep-layer – but not by late-born upper-layer – neurons (**Figures 3A–D** and Supplementary Figures 4A–D) (Hevner et al., 2001; Englund et al., 2005; Molyneaux et al., 2007; Toma and Hanashima, 2015). Quantification of the CP thickness revealed that corticogenesis in the tree shrew starts before E32 and is largely completed at the time of birth, with E32 representing the stage at which mainly Tbr1+ deep-layers are being produced and E37 the stage at which largely Tbr2– upper-layers are being produced (**Figure 3E**). Double-immunofluorescence for Brn2, characteristically expressed by upper-layer neurons, and Tbr1 revealed that a major fraction of Tbr1– cells in the E37–P1 CP belong to the population of upper-layer neurons, thus corroborating E32 to represent the peak of deep-layer and E37 the peak of upper-layer production (Supplementary Figures 4E–H) (McEvilly et al., 2002; Sugitani et al., 2002; Britanova et al., 2008; Glatzle et al., 2017). Together, this indicates that – similar to other mammalian species – the SVZ of the developing tree shrew neocortex is an important site of neurogenesis, being largest at developmental stages when upper layers of the CP are being produced (Kriegstein et al., 2006; Kriegstein and Alvarez-Buylla, 2009).

### Pax6+ NPCs Are Markedly Increased at Peak of Upper-Layer Neurogenesis in the Tree Shrew Neocortex

We next focused our analysis on the characterization of the distinct NPCs in the tree shrew neocortex, and analyzed E32–P1 cortical sections by double-immunofluorescence for the expression of the NPC markers Pax6, a transcription factor characteristically expressed by APs and bRG, and Tbr2, a transcription factor characteristically expressed by bIPs (**Figures 4A–D**) (Englund et al., 2005; Fietz et al., 2010; Hansen et al., 2010; Reillo et al., 2011). At all stages analyzed, the overwhelming majority of Tbr2+ (Pax6–/Tbr2+, Pax6+/Tbr2+) NPCs is present in the tree shrew SVZ, as observed in most mammalian species (**Figures 4A–F,H**). At peak of upper-layer neurogenesis, i.e., E37, when iSVZ and oSVZ were clearly distinguishable from each other, a large proportion of Tbr2+ NPCs resides in the iSVZ. However, the majority of Tbr2+ NPCs is distributed in the tree shrew oSVZ, which is similar to the developing macaque neocortex but different to that of ferret and rat, in which the majority of Tbr2+ NPCs remained in the iSVZ (**Figures 4B,J**) (Martinez-Cerdeno et al., 2012).

In line with observations in other mammalian species (Götz et al., 1998; Englund et al., 2005; Osumi et al., 2008), NPCs of the tree shrew VZ were Pax6+ and largely Tbr2– with the number of Pax6+/Tbr2– NPCs progressively declining during development (**Figures 4A–E**). At E32 the number of Pax6+ (Pax6+/Tbr2–, Pax6+/Tbr2+) NPCs was relatively small in the tree shrew SVZ (**Figures 4A,F**), thus revealing Pax6+ NPCs to be predominantly distributed in the tree shrew VZ at early stages of neocortex development (**Figure 4G**). However, at later developmental stages, especially at peak of upper-layer neurogenesis, i.e., E37, the number of Pax6+ (Pax6+/Tbr2–, Pax6+/Tbr2+) NPCs markedly increases in the tree shrew SVZ (**Figures 4B–D,F**) shifting the distribution of Pax6+ (Pax6+/Tbr2–, Pax6+/Tbr2+) NPCs to the SVZ, especially the oSVZ (**Figures 4G,I**). This is in line with findings in the developing neocortex of macaque and human, but in contrast to those reported for ferret and rat, in which the majority of Pax6+ NPCs remained in the VZ until the end of neurogenesis (Bayatti et al., 2008; Fish et al., 2008; Fietz et al., 2010; Hansen et al., 2010; Martinez-Cerdeno et al., 2012; Betizeau et al., 2013). Unlike the majority of Tbr2+ NPCs, which also expressed Pax6+ (**Figures 4F,H**), the vast majority of Pax6+ NPCs in the tree shrew SVZ were Tbr2– with the highest abundance of NPCs exclusively expressing Pax6 being present at E37 (**Figures 4F,G**). This raises the possibility that – similar to the developing neocortex of primates and other species exhibiting an expanded neocortex such as ferret – the tree shrew SVZ contains bRG at high abundance at peak of upper layer neurogenesis.

### High Relative Abundance of bRG at Peak of Upper-Layer Neurogenesis in the Tree Shrew Neocortex

To examine whether Pax6+/Tbr2– NPCs in the tree shrew SVZ extend radially oriented processes at M-phase, and thus represent bRG, we analyzed E37 cortical sections by immunohistochemistry for triple immunofluorescence for Pax6, Tbr2 and phosphorylated vimentin (pVim), which visualizes cellular processes of mitotic NPCs (**Figures 5A–F**). This revealed that apically dividing Pax6+ NPCs in the tree shrew VZ, i.e., APs, exhibit a radially oriented process (**Figure 5A**). Moreover, the tree shrew SVZ not only comprises mitoses that lack processes of any substantial length, i.e., bIPs, with a large number of them being Tbr2+ (**Figures 5B,L**), but also mitoses that exhibit at least one well-developed radially oriented process, i.e., bRG, with the majority of them expressing Pax6 but not Tbr2 (**Figures 5C–E,K**).

Intriguingly, also the majority of dividing bIPs in the developing tree shrew SVZ were found to be Pax6+ (**Figure 5L**). A relatively high abundance of Pax6+ bIPs has been described for the developing macaque SVZ, but not for that of mouse or rat (Betizeau et al., 2013).

None of the apical processes extending from the dividing bRG reached the ventricular surface (**Figures 5D,E**). Moreover, Par3, a protein associated with the apical cell cortex (Manabe et al., 2002; Costa et al., 2008) and ZO-1, a protein associated with apical adherens junctions (Aaku-Saraste et al., 1996), were highly concentrated at the tree shrew ventricular surface, but not in the tree shrew SVZ (**Figure 6**) indicating that tree shrew bRG lack an apical domain. Importantly, the relative bRG abundance in the tree shrew SVZ was much higher than that obtained in the developing SVZ of lissencephalic rodents; however, slightly smaller than that obtained in the developing SVZ of gyrencephalic primates including human and other species exhibiting an expanded neocortex including ferret (**Figure 5H**) (Fietz et al., 2010; Hansen et al., 2010; Reillo et al., 2011; Wang et al., 2011; Betizeau et al., 2013). Moreover, similar to the developing macaque neocortex, we observed three bRG morphologies in the tree shrew SVZ (Betizeau et al., 2013): bRG exhibiting a basal process, referred to as bRG-basal-process (-P), bRG exhibiting an apical process, referred to as bRG-apical-P and bRG exhibiting both, a basal and an apical process, referred to as bRG-both-P with the overwhelming majority of tree shrew bRG representing bRG-basal-P (**Figure 5I**). Interestingly, a small percentage of NPCs in the tree shrew E37 SVZ exhibited one or two tangentially oriented processes, i.e., processes that are oriented parallel to the ventricular surface at mitosis (**Figures 5F,H**), previously referred to as basal tangential glia (bTG) (Sauerland et al., 2018). This cell type has been shown to be present in the developing macaque SVZ (Betizeau et al., 2013) in a similar abundance; however, appears to be absent in the developing rodent SVZ.

Taken together, our findings reveal that the developing tree shrew SVZ consists of at least three main NPCs: the process-lacking bIP and the process-bearing bRG and bTG, all exhibiting similar cell biological features including location of mitosis, process retention and direction at mitosis, and molecular marker expression as has been described for gyrencephalic primates. Importantly, the tree shrew neocortex exhibits a relatively high relative abundance of bRG at peak of upper-layer neurogenesis. In conclusion, our data reveal that key features of tree shrew neocortex development, e.g., the development of the germinal zones and the distribution and abundance of distinct NPCs appear to show a closer relationship to gyrencephalic primates than to lissencephalic rodents.

### Key Features of Tree Shrew Neocortex Development Are Closer Related to Those of Gyrencephalic Primates Than to Those of Lissencephalic Rodents

**FIGURE 7:**
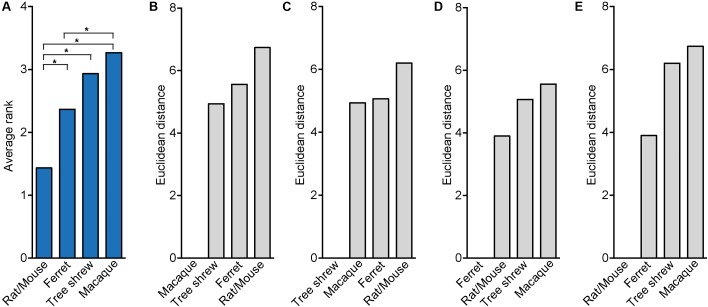
Rank test and Euclidean distance computation illustrating the relationship between tree shrew, macaque, ferret and rat/mouse neocortex development. **(A)** Bar plot showing the average ranks, which were obtained over all neurodevelopmental parameters of tree shrew, macaque, ferret and rat/mouse (**Table 2**). Statistical differences between the average ranks of the different species were tested with the Kruskal–Wallis-test followed by Conover’s *post hoc* test using Holm correction. Asterisks indicate significant *P*-values (*P* < 0.05). **(B–E)** Bar plots by Euclidean distance computation using z-transformed values of distinct parameters of tree shrew, macaque, ferret and rat/mouse neocortex development (**Table 2**). **(A–E)** For details, see Section “Materials and Methods.”

To quantify the strength of the relationship between neocortex development of the tree shrew and phylogenetically closely related species, we obtained distinct neurodevelopmental parameters of the tree shrew, a gyrencephalic primate (i.e., macaque), a lissencephalic rodent (i.e., rat, mouse) and, for comparison, a gyrencephalic carnivore (i.e., ferret) that exhibits an encephalization quotient similar to the tree shrew (Supplementary Figure 1B). We first used a rank test and compared the average ranks, which were obtained over all neurodevelopmental parameters for each species, between the four different species (**Table 2** and **Figure 7A**). This revealed that the average rank of mouse/rat is significantly different when compared to that of all other species and most similar to that of ferret, indicating that the pattern of neurodevelopmental parameters of mouse/rat is clearly distinct from ferret, tree shrew and macaque and more similar to that of ferret than to that of tree shrew and macaque (**Figure 7A**). Intriguingly, the average rank of tree shrew differs significantly from that of mouse/rat, but not from that of ferret and macaque, suggesting that the pattern of tree shrew neurodevelopmental parameters is closer related to that of macaque and ferret than to that of mouse/rat (**Figure 7A**). As the smallest difference in the average rank has been observed between the tree shrew and the macaque, neurodevelopmental parameters of tree shrew appear to be closest related to those of macaque (**Figure 7A**).

**FIGURE 8:**
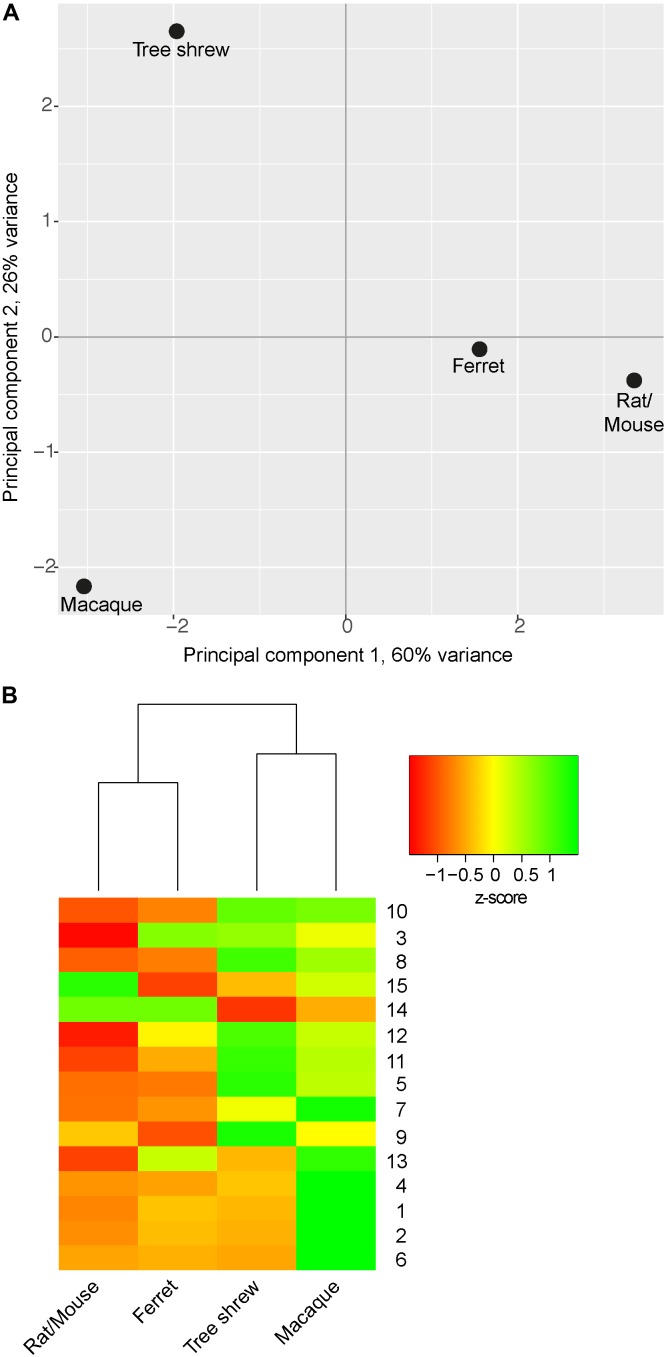
Principal component analysis (PCA) and hierarchical clustering illustrating the relationship between tree shrew, macaque, ferret and rat/mouse neocortex development. **(A)** Cluster plot by PCA using z-transformed values of distinct parameters of tree shrew, macaque, ferret and rat/mouse neocortex development (**Table 2**). **(B)** Heatmap showing hierarchical clustering of z-transformed values of distinct parameters of neocortex development between tree shrew, macaque, ferret and rat/mouse (**Table 2**). Hierarchical clustering of columns was performed based on Euclidean distance. Rows were sorted in order of increasing distance between the parameters of tree shrew and macaque from top to bottom. Row numbers refer to neurodevelopmental parameters as listed in **Table 2**. UL, upper layer; DL, deep layer. **(A,B)** For details, see Section “Materials and Methods.”

In a next step, neurodevelopmental parameters of tree shrew, macaque, rat/mouse and ferret were compared by calculating pair-wise Euclidean distances between them (**Figures 7B–E**). This showed that the shortest Euclidean distance, and thus the highest similarity, was observed between neurodevelopmental parameters of rat/mouse and ferret (**Figures 7D,E**). Neurodevelopmental parameters of the tree shrew exhibited the shortest Euclidean distance, and thus the highest similarity, to those of macaque, and a shorter Euclidean distance to those of ferret than to that of rat/mouse (**Figure 7C**). Moreover, principal component (PC) analysis using the neurodevelopmental parameters of tree shrew, macaque, rat/mouse and ferret revealed that on the first PC, which explains about two-thirds of the total variance, the score depicting tree shrew neocortex development falls closest to that of macaque and more closer to that of ferret than to that of rat/mouse, indicating that tree shrew neocortex development is most similar to that of macaque and more similar to that of ferret than to that of rat/mouse (**Figure 8A**).

The results were corroborated when the parameters of tree shrew, macaque, rat/mouse and ferret neocortex development were clustered hierarchically with single parameters being sorted in order of increasing distance between tree shrew and macaque (**Figure 8B**). Again, this showed that neurodevelopmental parameters of rat/mouse and ferret are most closely related to each other and that parameters of tree shrew neocortex development are most closely related to those of macaque. Among the parameters showing the highest resemblance between tree shrew and macaque neocortex development were: the ratio of Pax6+ NPCs in SVZ/VZ and the relative abundance of Pax6+ bRG at upper-layer neurogenesis, both being clearly distinct from neocortex development of rodents (**Figure 8B**). Together, the results of the statistical analysis of the relationship between the tree shrew and related species indicate that key features of tree shrew neocortex development are most closely related to those of gyrencephalic primates and more closely related to those of ferret than to those of lissencephalic rodents.

## Discussion

This study provides – for the first time – detailed data on the presence, abundance and distribution of the distinct NPCs in the developing neocortex of the northern tree shrew (*Tupaia belangeri*). We show that – similar to gyrencephalic primates – the developing tree shrew neocortex is characterized by an expanded SVZ, a high abundance of Pax6+ NPCs in the SVZ, and a relatively high percentage of bRG at peak of upper-layer neurogenesis. Using rank comparison, Euclidian distance computation, PCA and hierarchical clustering, this study provides first evidence that key features of tree shrew neocortex development, i.e., the development of the germinal zones and the distribution and abundance of distinct NPCs, are indeed closer related to those of gyrencephalic primates than to those of ferret and lissencephalic rodents. Hence, our results support the hypothesis that physiological, neuroanatomical and developmental characteristics of the tree shrew are very closely related to primates (Sur et al., 1980; Elston et al., 2005; Remple et al., 2007; Wong and Kaas, 2009a; Veit et al., 2011; Knabe and Washausen, 2015; Xiao et al., 2017; Yao, 2017).

Given that the tree shrew possesses a lissencephalic neocortex, our study provides further evidence for the notion that the abundant occurrence of bRG is not exclusively linked to the development of a gyrencephalic neocortex (Garcia-Moreno et al., 2012; Kelava et al., 2012; Lewitus et al., 2013). Moreover, our statistical analysis reveals neurodevelopmental parameters of tree shrew to be closest related to gyrencephalic macaque and those of gyrencephalic ferret to be closest related to lissencephalic rat/mouse, thus indicating that the abundance and distribution of distinct NPCs seem not to be correlated with the degree of cortical surface folding. In this regard, it would be interesting to unravel the relationship of tree shrew neocortex development to that of gyrencephalic rodents such as agouti and lissencephalic primates such as marmoset in more depth. However, as the available data of neocortex development of gyrencephalic rodents and lissencephalic primates are limited and currently not matched to neurogenic stages, we have not included them in our statistical analysis (Garcia-Moreno et al., 2012; Kelava et al., 2012). Thus, further studies including a wider range of developmental stages and neurodevelopmental parameters such as absolute values of NPC counts, cell cycle length and NPC division mode of more different mammalian species are necessary in order to gain a deeper understanding of the relationship between neocortex development of the tree shrew and phylogenetically closely related animals.

Interestingly, our data show that – although a large number of dividing bIPs in the developing tree shrew neocortex express Tbr2 – the overwhelming majority of them express Pax6. In this context it is interesting to note that the majority of bIPs in the developing macaque neocortex is able to proliferate and seems to sustain Pax6 expression whereas the majority of bIPs in the rat and mouse neocortex undergoes symmetric neurogenic division and downregulates Pax6 (Betizeau et al., 2013). It might therefore be speculated that – similar to gyrencephalic primates – the majority of bIPs in the tree shrew neocortex might belong to the proliferative subtype. Indeed, the rapid expansion of the SVZ as well as rapid increase of Pax6+ NPCs in the tree shrew SVZ between E32–37, which are accompanied by only a moderate decrease in VZ size, support the notion of self-amplifying NPCs being present in high abundance in the tree shrew SVZ. Moreover, our data indicate that the period of neurogenesis, specifically the period between peak of upper- and deep-layer neurogenesis, in the tree shrew neocortex is relatively short (approximately 5 days) being similar to that of rat (approximately 4 days) and much shorter when compared to that of ferret (approximately 12 days) (Workman et al., 2013). Hence, the high abundance of highly proliferative NPCs, i.e., proliferative bIPs and bRG, in the developing neocortex might enable the tree shrew to achieve an encephalization quotient similar to that of ferret and much higher when compared to that of rat, e.g., by producing more cells (neurons) per time unit. Further studies using long-term live cell imaging of NPCs in the developing tree shrew neocortex are needed in order to evaluate the proliferative potential and contribution of the distinct NPCs, specifically of bIPs and bRG, to tree shrew neocortex development.

The tree shrew has been receiving increasing attention as an experimental model organism for studying fundamental biological functions and disease mechanisms including viral and bacterial infections, cancer, metabolic diseases and central nervous system related functions and disorders such as psychosocial stress, learning, aging, acoustic communication, myopia and Alzheimer’s disease (Binz et al., 1990; Fuchs and Flügge, 2002; Cao et al., 2003; Fuchs et al., 2004; Schehka et al., 2007; Schehka and Zimmermann, 2009; Konerding et al., 2011; Yamashita et al., 2012; Xiao et al., 2017; Yao, 2017). Because of the close phylogenetic and genetic relationship to primates, a short life span (6–8 years) and reproductive cycle (∼6 weeks), and high reproductivity with an average litter size of 2–3, the tree shrew has been proposed to replace primates in biomedical research (Norton and McBrien, 1992; Cao et al., 2003; Xiao et al., 2017; Yao, 2017). Moreover, the release of a publicly available annotated tree shrew genome sequence, the recent development of tree shrew transgenic technology and the current establishment of tree shrew inbred lines has greatly consolidated the position of the tree shrew in the field of disease animal models (Fan et al., 2013; Li et al., 2017; Yao, 2017). Until now, rodents, i.e., rat and mouse, have been most commonly used to model human brain development and to study developmental brain disorders (Florio and Huttner, 2014; Fernandez et al., 2016). However, because of the differences in neocortex development, specifically in the abundance and distribution of the distinct NPCs, between rodent and human, alternative model organisms exhibiting NPC characteristics similar to humans are urgently needed in order to better model and understand human neocortex development and developmental disorders (LaMonica et al., 2012). As the use of non-human primates in biomedical research is ethically troubling, the search for non-primate model organisms, that reflect key aspects of human neocortex development, is warranted (Barnhill et al., 2016; DeGrazia, 2016; Andersen and Winter, 2017; Neuhaus, 2017). By demonstrating that the tree shrew shows NPC characteristics very similar to gyrencephalic primates including human, this study introduced a potential novel experimental organism as an alternative to primates, which is seemingly more suitable than ferret and rodents, to model and understand specific aspects of human neocortex developmental and developmental brain disorders, i.e., where neocortex size is affected such as microcephaly.

## Conclusion

This study reveals that the developing tree shrew neocortex is characterized by an expanded SVZ, a high abundance of Pax6+ NPCs in the SVZ, and a relatively high percentage of bRG at peak of upper-layer neurogenesis. Moreover, it provides first evidence that key features of tree shrew neocortex development, i.e., the development of the germinal zones and the distribution and abundance of distinct NPCs, show a closer relationship to those of gyrencephalic primates than to those of ferret and lissencephalic rodents. Together, our data provides novel insight into the evolution of bRG and other distinct NPCs in the neocortex development of Euarchontoglires and introduces the tree shrew as a potential novel model organism in the area of human brain development and developmental disorders.

## Author Contributions

SR and SF: conceived and designed the experiments. SR, HB, WK, EZ, RR, and JS: performed the experiments and/or contributed materials. SR, WK, and SF: analyzed the data. SR, WK, EZ, RR, JS, and SF: discussed the data. SR, WK, and SF: wrote the article. All authors reviewed and approved the manuscript.

## Conflict of Interest Statement

The authors declare that the research was conducted in the absence of any commercial or financial relationships that could be construed as a potential conflict of interest.
