# Supplementary material for: Neural Progenitors in the Developing Neocortex of the Northern Tree Shrew (Tupaia belangeri) Show a Closer Relationship to Gyrencephalic Primates Than to Lissencephalic Rodents
Source: Front Neuroanat. 2018 Apr 19;12:29. doi: 10.3389/fnana.2018.00029 (PMC5917011; doi:10.3389/fnana.2018.00029)
Supplement: Supplementary file 1 [file Image_1.PDF]

Supplementary Material

Neural Progenitors in the Developing Neocortex of the Northern Tree Shrew (*Tupaia belangeri*) Show a Closer Relationship to Gyrencephalic Primates than to Lissencephalic Rodents

Sebastian Römer, Hannah Bender, Wolfgang Knabe, Elke Zimmermann, Rudolf Rübsamen, Johannes Seeger and Simone A. Fietz*

*Correspondence: simone.fietz@vetmed.uni-leipzig.de

Supplementary Figures

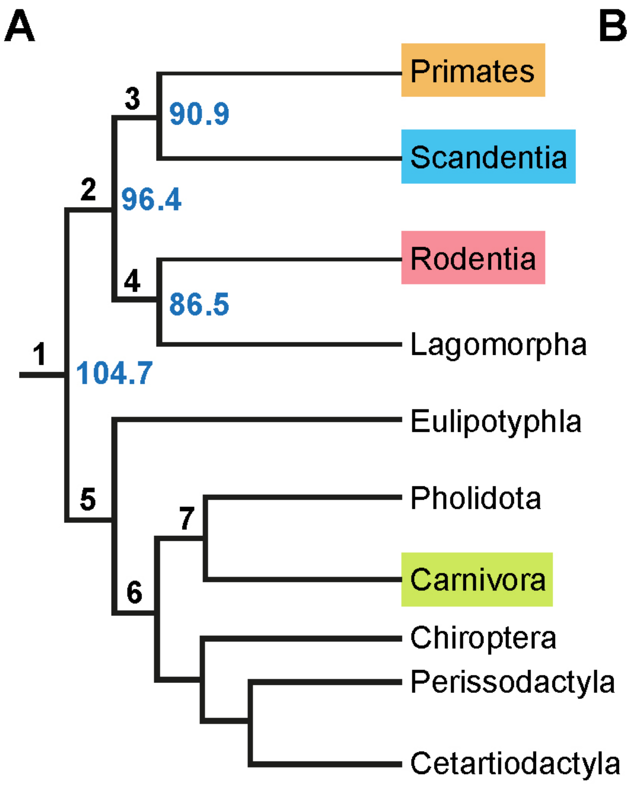

B

Marmoset	Macaque	Human	
288.12	7102.83	61770.32	BoW
7.61	89.22	1300	BrW
1.76	2.9	5.72	EQ
1.17	1.79	2.56	GI
146	166	275	GL

Tree shrew	
173.33	BoW
3.03	BrW
1.05	EQ
1.06	GI
43.7	GL

Mouse	Rat	Agouti	
40.4	315.1	2843.3	BoW
0.42	1.8	18.37	BrW
0.53	0.45	0.91	EQ
1.03	1.02	1.23	GI
21	22	110	GL

Ferret	
809	BoW
8.25	BrW
0.87	EQ
1.75	GI
42	GL

SUPPLEMENTARY FIGURE 1 | Relationship of the tree shrew to other mammalian orders with respect to the position in the phylogenetic tree and key physiological parameters. **(A)** Cladogram of the magnorder Boreoeutheria showing the phylogenetic relationship between the order Scandentia and closely related taxons. Numbers in black refer to the following taxons: 1, Boreoeutheria; 2, Euarchontoglires; 3, Euarchonta; 4, Glires; 5, Laurasiatheria; 6, Scrotifera; 7, Ferae. Data were obtained from the literature (Fan et al., 2013; Kumar et al., 2013; O'Leary et al., 2013). Numbers in blue refer to the possible divergence time in million years (Fan et al., 2013). Please note that the order Dermoptera is not depicted. Previous studies have placed it with the primates (Janecka et al., 2007) or with Scandentia (Murphy et al., 2001; Graphodatsky et al., 2011). **(B)** Physiological parameters of representative species, i.e. marmoset, macaque, human, tree shrew, mouse, rat, agouti, ferret of the orders Primates (orange), Scandentia (blue), Rodentia (red) and Carnivora (green), respectively. BoW, adult body weight (g); Brw, adult brain weight (g); EQ, encephalization quotient; GI, gyrification index; GL, gestation length (days). Data were obtained from the literature: BoW (Herculano-Houzel, 2007; Lewitus et al., 2014), BrW (Herculano-Houzel, 2007; Lewitus et al., 2014), EQ (Boddy et al., 2012; Steinhausen et al., 2016), GI (Pillay and Manger, 2007; Lewitus et al., 2014) and GL (Kuhn and Schwaier, 1973; Barton and Capellini, 2011; Garcia-Moreno et al., 2012; Lewitus et al., 2014).

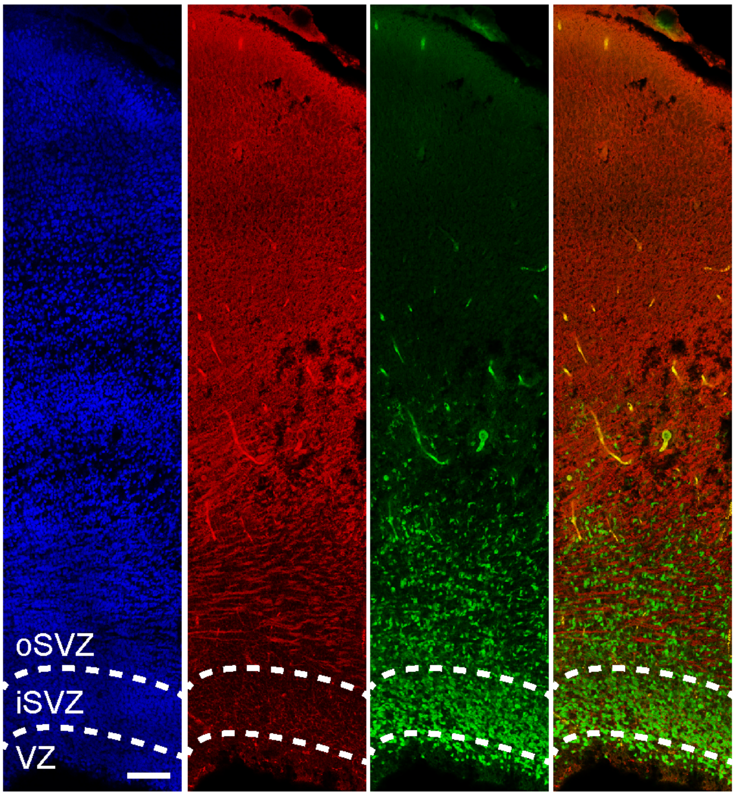

SUPPLEMENTARY FIGURE 2 | Identification of VZ, iSVZ and oSVZ in the developing tree shrew neocortex. Double-Immunofluorescence for Tau1 (red) and Tbr2 (green) and DAPI staining (blue) on 30 µm-cryosections of E37 tree shrew neocortex. Upper dashed lines indicate the boundary between iSVZ and oSVZ. Lower dashed lines indicate the boundary between VZ and iSVZ. For details, see Materials and Methods. VZ, ventricular zone; iSVZ, inner SVZ; oSVZ, outer SVZ. Scale bars, 100 µm.

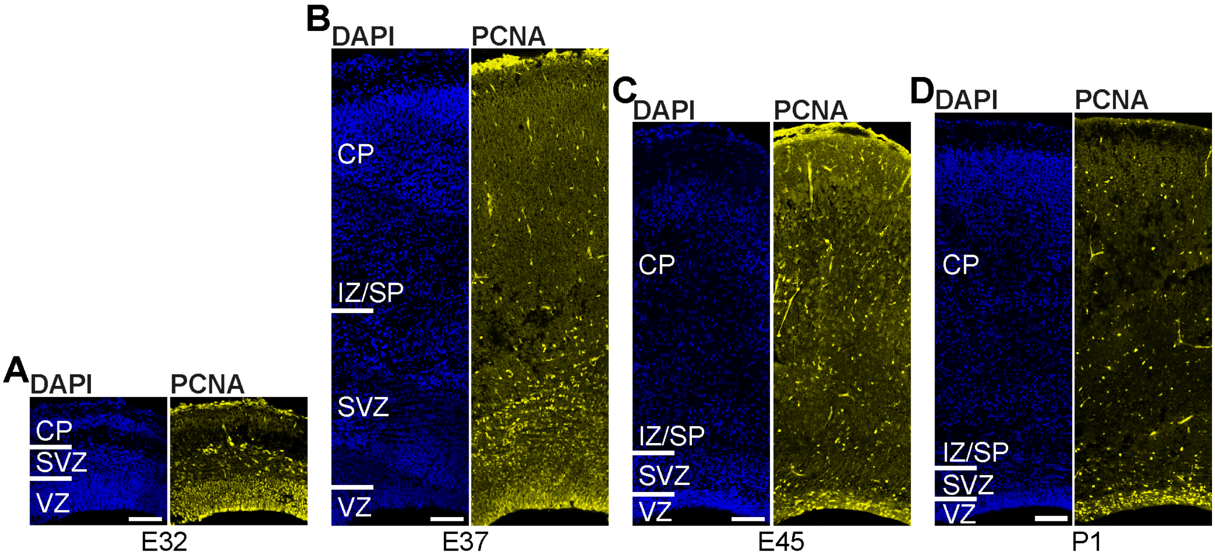

SUPPLEMENTARY FIGURE 3 | Development of the germinal zones with respect to the size of the cortical wall in the tree shrew neocortex. **(A–D)** Immunofluorescence for PCNA (yellow) and DAPI staining (blue) on 30 µm-cryosections of E32–P1 tree shrew neocortex. Scale bars, 100 µm. VZ, ventricular zone; SVZ, subventricular zone; IZ, intermediate zone; SP, subplate; CP, cortical plate.

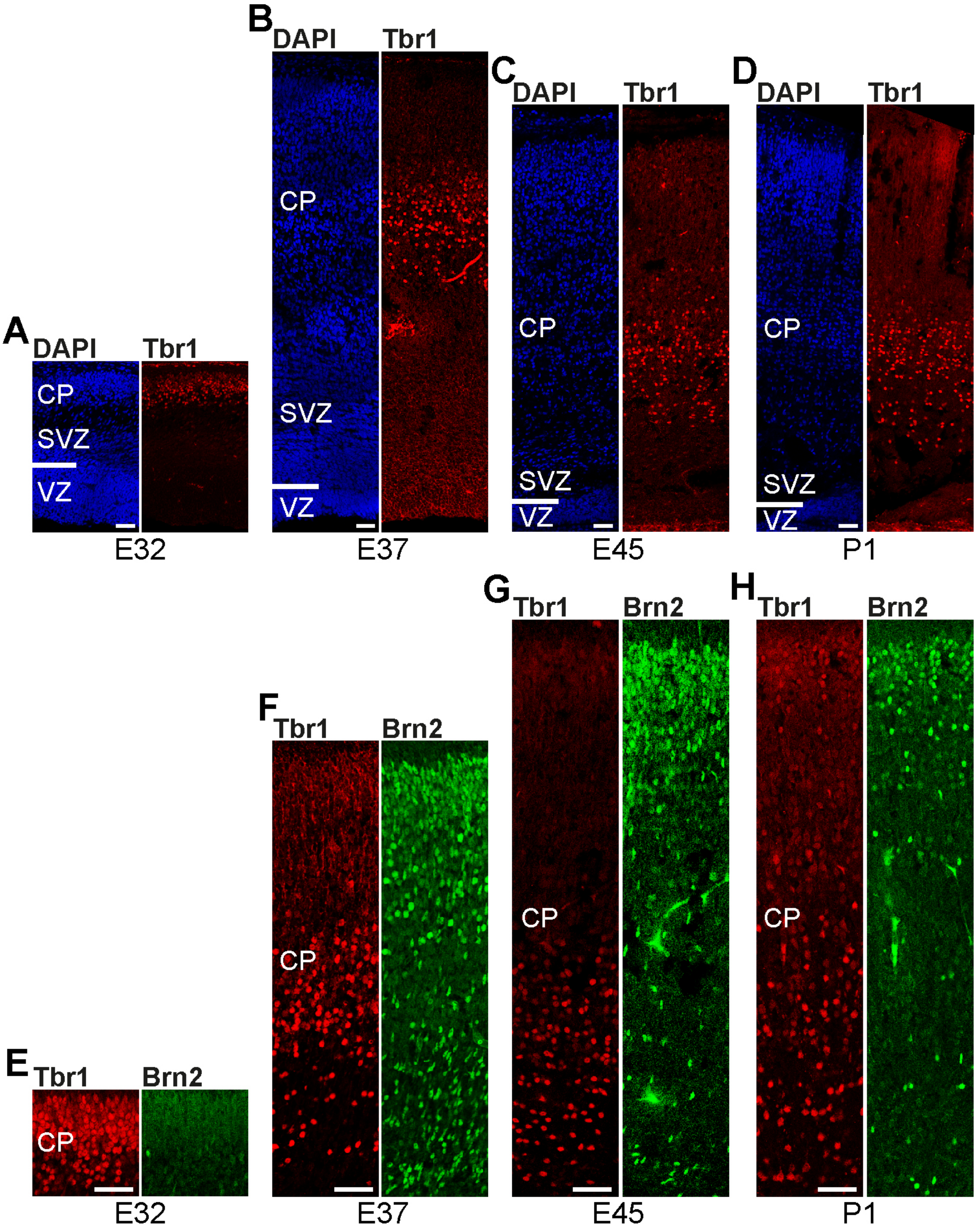

SUPPLEMENTARY FIGURE 4 | Development of the cortical plate with respect to the size of the cortical wall in the tree shrew neocortex. **(A–D)** Immunofluorescence for Tbr1 (red) and DAPI staining (blue) on 30 µm-cryosections of E32–P1 tree shrew neocortex. **(E–H)** Double-immunofluorescence for Tbr1 (red) and Brn2 (green) on 30 µm-cryosections of E32–P1 tree shrew neocortex. The bottom margin corresponds to the transition zone CP/subplate**. (A–H)** Scale bars, 50 µm. VZ, ventricular zone; SVZ, subventricular zone; CP, cortical plate.

References

Barton, R.A., and Capellini, I. (2011). Maternal investment, life histories, and the costs of brain growth in mammals. *Proc. Natl. Acad. Sci. U. S. A.* 108**,** 6169-6174. doi: 10.1073/pnas.1019140108

Boddy, A.M., Mcgowen, M.R., Sherwood, C.C., Grossman, L.I., Goodman, M., and Wildman, D.E. (2012). Comparative analysis of encephalization in mammals reveals relaxed constraints on anthropoid primate and cetacean brain scaling. *J. Evol. Biol.* 25**,** 981-994. doi: 10.1111/j.1420-9101.2012.02491.x

Fan, Y., Huang, Z.Y., Cao, C.C., Chen, C.S., Chen, Y.X., Fan, D.D., et al. (2013). Genome of the Chinese tree shrew. *Nat. Commun.* 4**,** 1426. doi: 10.1038/ncomms2416

Garcia-Moreno, F., Vasistha, N.A., Trevia, N., Bourne, J.A., and Molnar, Z. (2012). Compartmentalization of cerebral cortical germinal zones in a lissencephalic primate and gyrencephalic rodent. *Cereb. Cortex* 22**,** 482-492. doi: 10.1093/cercor/bhr312

Graphodatsky, A.S., Trifonov, V.A., and Stanyon, R. (2011). The genome diversity and karyotype evolution of mammals. *Mol Cytogenet* 4**,** 22. doi: 10.1186/1755-8166-4-22

Herculano-Houzel, S. (2007). Encephalization, Neuronal Excess, and Neuronal Index in Rodents. *The Anatomical Record: Advances in Integrative Anatomy and Evolutionary Biology* 290**,** 1280-1287. doi: 10.1002/ar.20598

Janecka, J.E., Miller, W., Pringle, T.H., Wiens, F., Zitzmann, A., Helgen, K.M., et al. (2007). Molecular and genomic data identify the closest living relative of primates. *Science* 318**,** 792-794. doi: 10.1126/science.1147555

Kuhn, H.J., and Schwaier, A. (1973). Implantation, early placentation, and the chronology of embryogenesis in Tupaia belangeri. *Z. Anat. Entwickl. Gesch.* 142**,** 315-340. doi: 10.1007/BF00519135

Kumar, V., Hallstrom, B.M., and Janke, A. (2013). Coalescent-based genome analyses resolve the early branches of the euarchontoglires. *PLoS One* 8**,** e60019. doi: 10.1371/journal.pone.0060019

Lewitus, E., Kelava, I., Kalinka, A.T., Tomancak, P., and Huttner, W.B. (2014). An adaptive threshold in mammalian neocortical evolution. *PLoS Biol.* 12**,** e1002000. doi: 10.1371/journal.pbio.1002000

Murphy, W.J., Eizirik, E., O'brien, S.J., Madsen, O., Scally, M., Douady, C.J., et al. (2001). Resolution of the early placental mammal radiation using Bayesian phylogenetics. *Science* 294**,** 2348-2351. doi: 10.1126/science.1067179

O'leary, M.A., Bloch, J.I., Flynn, J.J., Gaudin, T.J., Giallombardo, A., Giannini, N.P., et al. (2013). The placental mammal ancestor and the post-K-Pg radiation of placentals. *Science* 339**,** 662-667. doi: 10.1126/science.1229237

Pillay, P., and Manger, P.R. (2007). Order-specific quantitative patterns of cortical gyrification. *European Journal of Neuroscience* 25**,** 2705-2712. doi: 10.1111/j.1460-9568.2007.05524.x

Steinhausen, C., Zehl, L., Haas-Rioth, M., Morcinek, K., Walkowiak, W., and Huggenberger, S. (2016). Multivariate Meta-Analysis of Brain-Mass Correlations in Eutherian Mammals. *Front. Neuroanat.* 10**,** 91. doi: 10.3389/fnana.2016.00091
